# Metabolic Symbiosis in Chemoresistance: Refocusing the Role of Aerobic Glycolysis

**DOI:** 10.3389/fonc.2020.00005

**Published:** 2020-01-24

**Authors:** Lisi Ma, Xiangyun Zong

**Affiliations:** Department of Breast Surgery, Shanghai Jiao Tong University Affiliated Sixth People's Hospital, Shanghai, China

**Keywords:** chemoresistance, mTOR, aerobic glycolysis, OXPHOS, metabolic symbiosis

## Abstract

Cellular metabolic reprogramming is now recognized as a hallmark of tumors. Altered tumor metabolism determines the malignant biological behaviors and phenotypes of cancer. More recently, studies have begun to reveal that cancer cells generally exhibit increased glycolysis or oxidative phosphorylation (OXPHOS) for Adenosine Triphosphate(ATP)generation, which is frequently associated with drug resistance. The metabolism of drug-resistant cells is regulated by the PI3K/AKT/mTOR pathway which ultimately confer cancer cells drug resistance phenotype. The key enzymes involved in glycolysis and the key molecules in relevant pathways have been used as targets to reverse drug resistance. In this review, we highlight our current understanding of the role of metabolic symbiosis in therapeutic resistance and discuss the ongoing effort to develop metabolic inhibitors as anti-cancer drugs to overcome drug resistance to classical chemotherapy.

## Cancer Cell Metabolism

Cancer cell metabolism plays an important role in the malignant biological behaviors of cancer, favoring cancer cell survival, proliferation, invasion, and metastasis. In 2011, Weinberg summarized cancer metabolism as one of the ten characteristics of cancers and proposed that aberrant metabolic patterns are closely associated with the malignant biological behavior of cancer (1, 2). In 1924, Otto Warburg first observed the anomalous traits of cancer metabolism. Tumor cells use glycolysis, even in the presence of oxygen. This state was termed “Warburg effect,” and it has been considered a universal phenotype of cancers ever since (1, 3). This became known as aerobic glycolysis which he interpreted as mitochondrial dysfunction. However, it is now clear that mitochondrial function is intact and essential for cancer cell viability (4). while Warburg effect is a necessary metabolic shift for cell division and proliferation, recent evidences suggests that mitochondria and OXPHOS are also essential to bioenergetics, biosynthesis, and signaling in proliferative and quiescent cancer cells such as cancer stem cells concentrated in drug-resistance tumors (5). Intriguingly, emerging studies have begun to demonstrate that cancer cell subsets with different dependencies in energy generating pathways coexist within tumors in a symbiotic manner (6).

## Mechanisms Underlying The Development of Cancer Cell Drug Resistance

**Figure 1:**
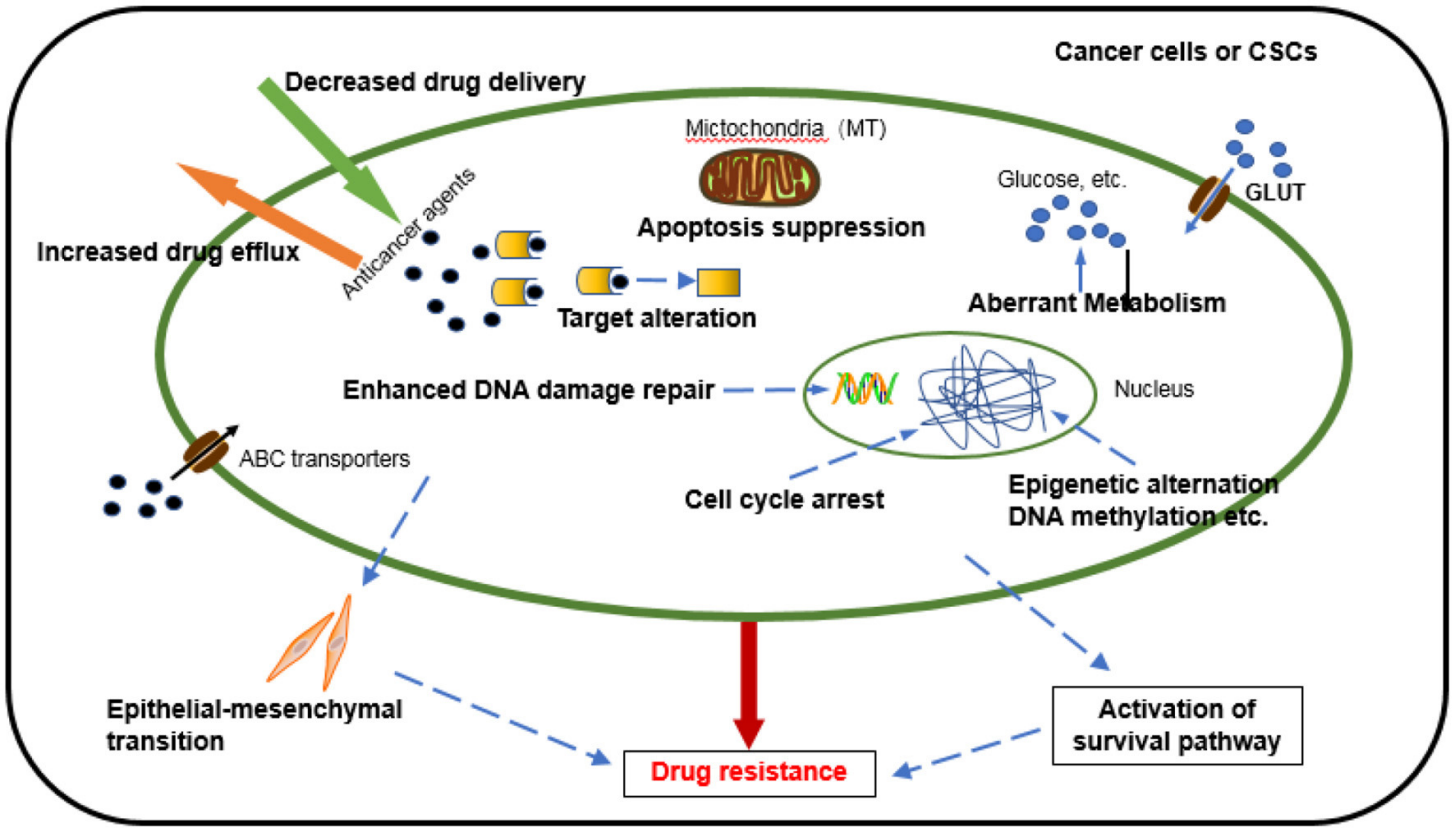
Mechanisms underlying the development of cancer cell drug resistance.

Chemotherapy, one of the dominant treatments in cancer therapy, has led to improvement in the quality of life and survival of cancer patients. Despite initially impressive clinical responses, most patients eventually develop a resistance to drugs. Chemoresistance can be manifested through various mechanisms (7) (Figure 1), including an alteration of drug metabolism due to increased efflux and decreased drug delivery, changes in drug targets, apoptosis suppression, activated intracellular survival signaling, enhanced DNA repair, epithelial-mesenchymal transition (EMT) mediated chemoresistance (8), immune escape of cancer stem cells promoting the development of chemoresistance, epigenetic alteration, and aberrant metabolism (9). Several new studies disclose that mitochondrial metabolism, in part, drives chemoresistance in cancer (10, 11). It is worth noting that these factors do not exist independently but interact and influence each other in many aspects to jointly regulate chemotherapy resistance (12). Although many mechanisms of tumor chemoresistance have been discovered and elucidated, it remains the leading cause of chemotherapy failure, especially in relapsed or metastatic tumors. Studies have shown that 90% of disease progression during or after chemotherapy is related to drug resistance (13). It is extremely urgent to conduct in-depth research on the mechanism of chemotherapy resistance and provide more effective strategies for clinical treatment (14).

Over the years, data have emerged indicating that aberrant cancer metabolism can also influence drug efficacy. Although increased aerobic glycolysis is accepted as a feature of tumors, its causal relationship with chemoresistance is still controversial. We postulate that metabolic rewiring, especially aerobic glycolysis and OXPHOS, are novel and important mechanism of drug resistance. This review will summarize current knowledge on the role of metabolic symbiosis in drug efficacy, how it contributes to chemoresistance, and how this information could be exploited to optimize cancer drug testing and cancer treatment.

## Altered Cancer Metabolism With Chemoresistance

### Metabolic Symbiosis in Cancer

Over the past years, the more prevalent metabolic reprograming described in tumor cells is an increase in glucose uptake, the enhancement of glycolytic capability and a high lactate production, along with the deficiency of respiration even in the presence of oxygen (Warburg effect). Nowadays, intense glycolysis is actually not observed in all tumor types, “aerobic-glycolysis” hypothesis has been opposed by an increasing number of studies indicating that mitochondria in tumor cells are not impaired but operate at low capacity (15). Furthermore, many investigations revealed contradictory alterations with the upregulation of OXPHOS elements and a larger dependence of cancer cells on oxidative energy substrates for anabolism and energy production (11, 16, 17). Griguer et al. displayed several glioma cell lines that were highly dependent on mitochondrial OXPHOS pathway to produce ATP (18), another glioma cells which utilize glycolysis can also switch from aerobic glycolysis to OXPHOS under limiting glucose conditions (19). As observed in other types of tumors, such as breast carcinoma, cervical cancer and pancreatic cancer. Tumor cells have different metabolic pathways under different culture conditions and microenvironments (20). The bioenergetic mode of a tumor switches between glycolytic and OXPHOS depending on tumor microenvironment and activated oncogenes. Aerobic glycolysis and OXPHOS cooperate during cancer progression (21). These effects may yield metabolic symbiosis (6), the hypothesis of metabolic symbiosis demands that the secreted lactate is not merely “toxic acidic waste,” but rather a source of fuel that is imported and metabolized by the normoxic cancer cells (22). The pattern of metabolic symbiosis provides a system by which cancer cells may adapt to or maximize the use of available resources, thereby increasing their capacity to grow and metastasize. Components of metabolic symbiosis, such as metabolic transporters and enzymes, may serve clinically as prognostic indicators in the future (23).

### Altered Metabolism Promotes Chemoresistance

Altered metabolism, especially the phenomenon of metabolic symbiosis, may confer cancer cells a chemoresistant phenotype (19, 22, 24). Cancer cells acquire drug resistance in the following ways: aberrant metabolism increasing energy production and drug efflux, the synthesis of metabolites, conferring the resistant phenotype to cancer stem cells, reducing drug-induced apoptosis, and activating proliferative signaling pathways. Mounting evidence suggests that drug-resistant cells overexpress energy-dependent ATP-binding cassette (ABC) transporters. This is because elevated ATP levels directly influence the activity of ABC transporters, which prevents the high accumulation of anti-cancer drugs in cells and promotes drug resistance. The inhibition of glycolysis or OXPHOS and deprivation of ATP can block the function of the efflux pump, thereby increasing the toxicity of the drug and reversing drug resistance. In addition, studies have shown that increased aerobic glycolysis results in lactic acid accumulation, acidification, and a hypoxic microenvironment, conferring a phenotype resistant to cancer stem cells. Increased glucose consumption creates a hypoglycemic microenvironment. Tumor cells regard this nutritional deficiency as a stress signal, which leads to activation of the stress signaling pathway to induce autophagy and escape apoptosis. Many molecules converge upstream of the metabolic pathway to activate the proliferative signaling pathway, synergistically regulate tumor metabolism, upregulate glycolysis activity, and glycolytic enzyme activity, and ultimately induce drug resistance. The substrate produced by the Warburg effect can be used in other metabolic pathways, such as fat synthesis, nucleotide synthesis, and amino acid synthesis, which are essential for cancer proliferation. Thus, altered metabolism confers adaptive, proliferative, and survival advantages on the drug-resistant cell.

## Targeting Aberrant Metabolism Overcomes Chemotherapy Resistance

### Targeting OXPHOS in Cancer Cells

Recent perception is oxidative phosphorylation remains a central energy source for most malignancies, and mitochondria drive important tumor traits, such as tumor repopulation after oncogene ablation drug resistance, cancer stem cell maintenance, and disease expansion (25). Increasing evidences point to a role of mitochondrial OXPHOS in cancer progression and chemoresistance (26). A study in breast cancer showed that OXPHOS was the predominant supplier of ATP in both quiescent and proliferative layers (27). Increased OXPHOS in ovarian cancer cells promotes cancer cell survival and proliferation (28). Matilda Lee demonstrated that upregulation of mitochondrial STAT3 increases mitochondrial complex I and II activity, and therefore OXPHOS. The upregulation of OXPHOS is resistance mechanism to TKI therapy, evident in work studying the metabolic reprogramming of persistent cancer stem cells. Treating oncogene addicted tumors with both primary pathway TKIs and OXPHOS inhibitors may therefore reverse resistance (17). ROS-low leukemia stem cells are highly reliant on OXPHOS, and shown to be deficient to employ glycolysis. It's efficient to eradicate resistant LSC populations by targeting OXPHOS (29). A recent study describe a new cisplatin resistance mechanism in NSCLC based on a metabolic reprogramming that is therapeutically exploitable through PGC-1α downregulation or OXPHOS inhibitors (30).

### Targeting Glycolysis in Cancer Cells

#### Glucose Transport

The GLUT family consists of 14 members (GLUT1-14 or SLC2A1-14) that facilitate the uptake of glucose through cell membranes and play a vital role in glycolysis (31). Many studies have shown that upregulation of GLUT1, GLUT3, and GLUT4 is associated with cancer resistance. Inhibition of GLUT sensitizes the anti-cancer effects of chemotherapeutic drugs to overcome drug resistance in hypoxia (32). Increased GLUT1 levels are associated with the activation of mTOR signaling, resulting in an increase in glycolysis and a reduction in autophagy (33). Cao et al. demonstrated that GLUT1 is overexpressed in primary colon and breast cancer tissues. Under hypoxic conditions, phloretin, a GLUT1 inhibitor that blocks glucose uptake and glycolysis, re-sensitized both types of cancer cells to daunorubicin's anti-cancer activity and apoptosis-inducing effects (34). Similarly, the downregulation of GLUT1 increased the sensitivity of head and neck cancer cells to cisplatin (35). Clinically, temozolomide as adjunctive chemotherapy for malignant glioma significantly improved the survival and quality of life of patients. Unfortunately, most patients eventually presented chemoresistance. Le Calve B et al. found that glioblastoma cells were resistant to temozolomide after long-term exposure, which was related to the upregulation of GLUT3 expression. Moreover, selectively targeting GLUT3 delayed the occurrence of acquired resistance (36). Multiple myeloma cells rely on GLUT4 activity to uptake glucose and maintain Mcl-1 levels, growth, and survival. Wei et al. reported that ritonavir, an HIV protease inhibitor, could reduce the expression of Mcl-1 and GLUT4, inhibit glucose consumption and cell proliferation, induce cell apoptosis, and increase the sensitivity of cancer cells to chemotherapeutic drugs (37, 38). Although the combination of other targeted therapy drugs with the well-designed GLUT inhibitors may also be an effective way to overcome drug resistance, GLUTs are also widely expressed in other important organs and cells, which makes their targeting inadequate (39). Therefore, improving the affinity and selectivity of GLUT inhibitors is an important direction for anti-cancer research.

#### Hexokinase (HK)

Glycolysis enzymes play a central role in promoting resistant phenotypes (40). HK2 is the first rate-limiting enzyme in the glycolytic pathway and is highly expressed in many tumors. HK2 inhibitors such as 2-deoxyglucose (2-DG), 3-bromopyruvate (3-BrPA), and lonidamine (LND) have been used in preclinical experiments. Zhao et al. showed that increased glycolysis leads to trastuzumab resistance, and the combination of trastuzumab and 2-DG can effectively inhibit the glycolysis activity of breast cancer *in vitro* and *in vivo*. The combination synergistically inhibits the growth of trastuzumab-resistant breast cancer cells (41). Nakano's study found that, in multiple myeloma cells, increased glycolysis provides ample ATP, which is an important factor in promoting chemotherapy-resistant phenotypes. The HK2 inhibitor 3-BrPA reduces ATP production and enhances drug accumulation by inactivating ABC transporters, thereby restoring the cytotoxic effects of doxorubicin. 3-BrPA combined with doxorubicin can significantly inhibit the growth of subcutaneous tumors in multiple myeloma mice (42). *In vitro* and *in vivo* studies have demonstrated the anticancer effects of 3-BP on hepatocellular carcinoma, and consequently, this drug has been approved by the FDA (43). Importantly, 3-BrPA was confirmed in xenograft models *in vivo* by more than 75% tumor growth inhibition. In addition, Min et al. found that the development of the tumor-resistance phenotype can be achieved by regulating HK2 via the AKT/mTOR pathway (44).

#### Pyruvate Kinase (PK)

PKM2 is a crucial glycolytic enzyme that catalyzes the final step in the glycolytic pathway, which plays a dominant role in tumor growth and metabolism (45). Recent studies have reported that PKM2 promotes cell proliferation and prevents apoptosis and is highly expressed in many tumors (46). It is indicated that the upregulation of PKM2 is associated with drug resistance. Studies have found that the glycolysis activity of drug-resistant hepatocellular carcinoma cell lines is significantly upregulated, which confirmed that PKM2 is the downstream target of microRNA-122. Overexpression of microRNA-122 inhibited the activity of PKM2, thus reversing the drug resistance of adriamycin, inducing the apoptosis of drug-resistant cells and increasing drug sensitivity. Dysregulated glycose metabolism leads to doxorubicin resistance, and the inhibition of glycolysis induced by microRNA-122 may be a strategy to overcome doxorubicin resistance (47). PKM2 overexpression is a key mechanism of the chemoresistance of advanced bladder cancer to cisplatin. Inhibition of PKM2 via RNAi or chemical inhibitors may be a highly effective approach to overcoming chemoresistance and improving the outcome of advanced BC *in vivo* and *in vitro* (48). Moreover, PKM2 expression has been inversely correlated with resistance to several chemotherapeutics, including cisplatin, and oxaliplatin in gastric and colorectal cancers, respectively (48–50), The molecular mechanism underlying this is BMF, serving as a possible target gene of PKM2 that is involved in the oxaliplatin response and resistance in colorectal cancer via non-glycolysis (49). This contradictory conclusion has aroused people's concern about this drug target, which still needs to be clearly verified in different tumors in the future.

#### Enolase, ENO

ENO1 and ENO2 are highly conserved cytoplasmic glycolytic enzymes that catalyze the conversion of 2-phosphoglycerate into phosphoenolpyruvate (51). *In vitro* and *in vivo* studies demonstrated that higher expression levels of ENO-1 in breast tumor tissues are related to clinical therapeutic resistance (52). Gastric cancer cells acquire a drug-resistant phenotype by increasing aerobic glycolysis, and the inhibition of glycolysis reverses the sensitivity of cancers to chemotherapy (53). Liu et al. studied the effect of ENO2 on the survival of acute lymphoblastic leukemia (ALL) cells and found that it has a strong oncogenic function. ENO2 overexpression promotes cell growth and tumor formation in Nod/Scid mice by upregulating various glycolysis-related genes such as GLUT-1, LDH, and PKM2. This increases the glycolysis rate and ultimately leads to glucocorticoid resistance (54). In contrast, ENO2 silencing significantly inhibited cell proliferation and restores sensitivity to glucocorticoids. These results revealed that ENO2 expression can be a biomarker for predicting clinical efficacy of chemotherapy and relapse in ALL. Patients with ALL who overexpress ENO2 are more likely to develop glucocorticoid resistance. Therefore, the development of new ENO inhibitors or antibodies is coming, these strategies may provide potential therapeutic targets for ALL, and provide new insights for targeted therapy of tumors.

#### Lactate Dehydrogenase (LDH)

LDHA is one of the major isoforms of LDH expressed in breast tissue, catalyzing the last step of the glycolytic process. LDHA plays a key role in the glycolysis, growth features, and tumor maintenance of breast cancer cells (55). Studies have reported for the first time that the overexpression of LDHA is essential for the development of Taxol resistance in cancer cells. Experiments have shown that increased expression of LDHA promotes Taxol resistance, and LDHA inhibitor oxalate restrains the glycolysis pathway and causes a re-sensitization to Taxol. The combination of paclitaxel and oxalate promotes apoptosis in breast cancer cells and significantly increases the inhibition of paclitaxel-resistant cell growth, which may be an effective strategy to overcome paclitaxel resistance (56). Fu's study revealed that cetuximab (CTX)-resistant Ewing's sarcoma cells displayed upregulated LDHA expression and accelerated glycolysis. In the experiment, Ewing's sarcoma was re-sensitized to CTX by knocking down LDHA or inhibiting LDHA with oxalate to reduce the glycolysis rate of drug-resistant cells. The combined application of CTX and oxalate showed a synergistic inhibition effect to inhibit the viability of CTX-resistant cells, suggesting that LDHA inhibition may be an effective sensitizer for the treatment of cancer resistance (57). Another recent study reported that LDHA is a direct target of miR-34a, and the inhibition of LDHA by miR-34a promotes the re-sensitization of 5-fluorouracil-resistant colon cancer cells (58).

#### Fructose-1,6-Bisphosphatase (FBP)

Previous studies have focused on catabolic glycolysis, but recent studies have found that FBP, acting as a rate-limiting enzyme that controls the conversion of fructose 1,6-diphosphate to fructose 6-phosphate, plays a key role in tumor initiation and progression in various cancers. More importantly, in the study of chemoresistance, the role of FBP also has captured attention (59). FBP has two subtypes in mammalian cells. FBP1 has been proven to be a regulatory enzyme for gluconeogenesis, but the physiological role of FBP2 is unclear. Compared with normal tissues, FBP1 mRNA levels were significantly decreased in cervical cancer cells, and the downregulation of FBP1 expression was closely related to tumor recurrence and tumor stage in cervical cancer patients. Experiments validated that the overexpression of FBP1 significantly inhibits tumor cell growth and restores the chemosensitivity of cervical cancer cells by suppressing the glycolytic process (60). Gemcitabine, a first-line treatment for pancreatic cancer, is a drug that cancer cells often develop a resistance to. Studies have shown that gemcitabine treatment activates the RAS/RAF/MAPK pathway, and the IQ motif containing GTPase-activating protein 1 (IQGAP1) is used as a scaffold protein of MAPK, which directly regulates RAF, MEK, and extracellular signal-regulated kinases (ERKs). In pancreatic cancer, FBP1 inhibits the activation of ERK by gemcitabine via inhibiting the IQGAP1/ERK1/2 signaling pathway independent of its enzymatic activity, thereby enhancing the anti-cancer effect of drugs and overcoming drug resistance (61). However, whether FBP1 exerts similar drug resistance in other types of tumors needs further clarification. More research is needed to confirm whether FBP1 antagonizes the Warburg effect by inhibiting the glycolytic pathway or suppresses the above pathway through non-enzymatic activity and finally achieves the effect of reversing drug resistance.

In summary, the inhibition of glycolysis can effectively target drug-resistant and clonal tumor cells with high metabolic status and interfere with their metabolism to restore the cytotoxic effects of chemotherapeutic drugs. These findings confirm the novel role of enhanced glycolysis activity in tumor cell growth and drug resistance.

## Abnormal Activation of Signaling Pathways With Drug Resistance

**Figure 2:**
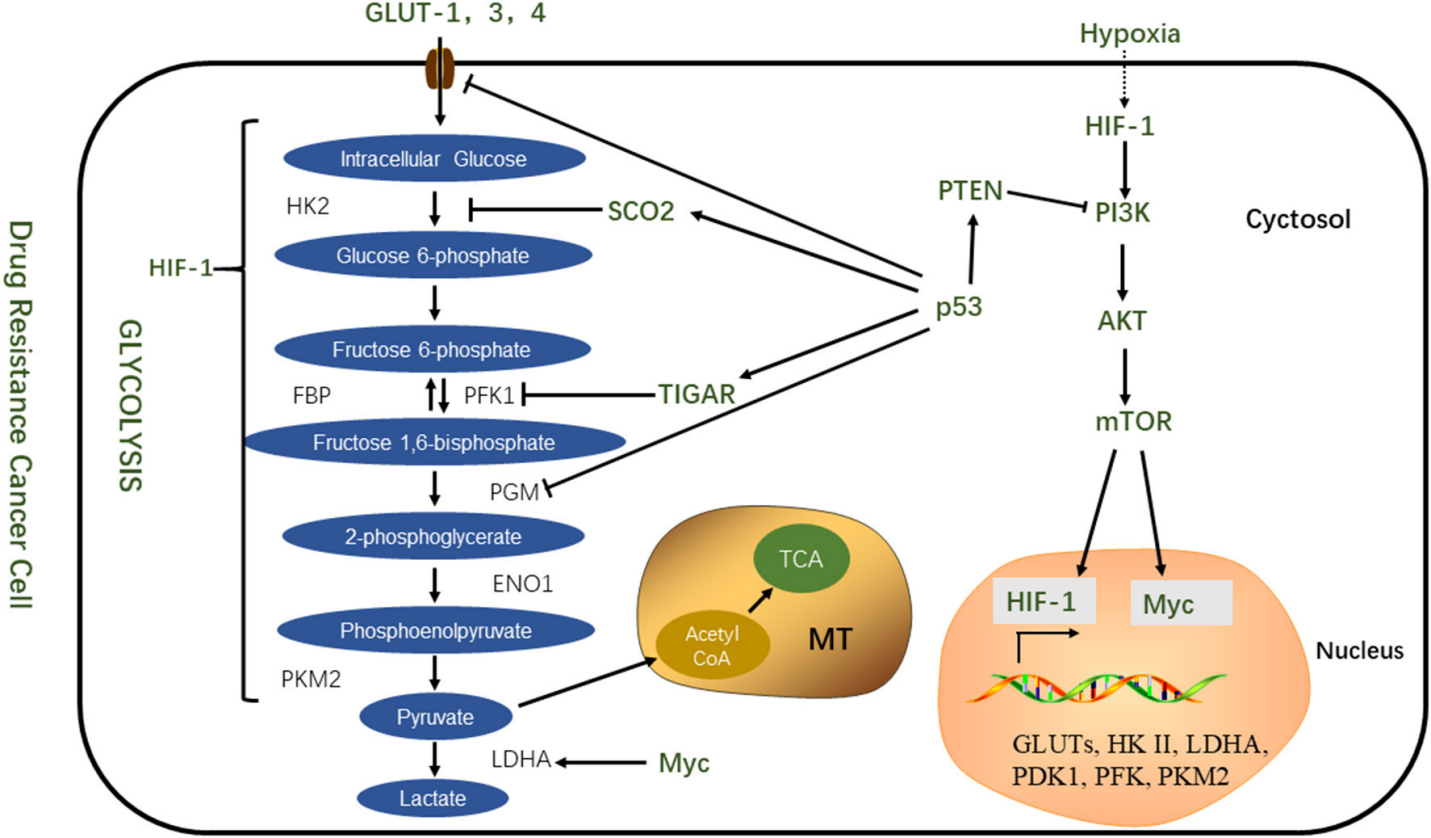
Transcription factors coordinate regulation of glycolytic metabolism.

The PI3K/AKT/mTOR pathway is not only involved in cell proliferation and apoptosis but also plays an important role in tumor proliferation and chemoresistance (62). Selective inhibition of the activity of this pathway can prevent the chemoresistance of tumor cells, and pathway inhibitors combined with chemotherapeutic drugs can enhance drug sensitivity. This suggests that the process of chemoresistance is correlated with the abnormal activation of the mTOR pathway (63). This pathway is activated by mutations in tumor suppressor genes such as PTEN, mutations in the PI3K complex component, or abnormal signaling of receptor tyrosine kinases. Once activated, the PI3K pathway not only provides a strong growth and survival signal for tumor cells but also has a profound effect on the metabolism of tumor cells (64). Downstream of PI3K is AKT1, an important driver of the tumor glycolytic phenotype, which stimulates ATP production through a variety of mechanisms to ensure that cells have sufficient bioenergy to respond to growth signals. AKT1 stimulates glycolysis, increases GLUT expression and membrane translocation, and phosphorylates key glycolytic enzymes (such as HK and FBP). Finally, AKT1 strongly stimulates the signal transduction of the mTOR pathway by phosphorylation and inhibition of its negative regulator, tuberous sclerosis 2 (TsC2). mTOR is a key metabolic integration site and a comprehensive control point for cell proliferation, growth and differentiation, nutrient uptake, and energy metabolism (65, 66). Activated mTOR stimulates protein and lipid synthesis and cell growth in response to adequate nutrient and energy conditions and is frequently activated during tumorigenesis (66). At the molecular level, mTOR directly stimulates mRNA translation and ribosomal biomacromolecular synthesis, and it indirectly induces other metabolic changes by activating transcription factors such as HIF1 under normoxic conditions (3) (Figure 2).

Zhang's experiments showed that doxorubicin multidrug-resistant cancer cells exhibit increased glucose intake and lactic acid production, and the activity of glycolytic enzymes also increase significantly; these included HK, PK, and LDH. In addition, GLUT4 was also overexpressed. The development of a resistant phenotype may be due to an over-activated AKT/mTOR/Myc signaling pathway. The target of the AKT/mTOR signaling pathway is a key regulator of glycometabolic homeostasis, which mediates the overexpression of c-Myc in drug-resistant cells and directly stimulates glucose uptake and enhances glycolysis. The inhibition of glycolysis can effectively kill sensitive and drug-resistant leukemia cells and effectively restore the susceptibility of drug-resistant cells to adriamycin. Therefore, inhibiting aerobic enzymolysis may be a potential therapeutic strategy for relapsed or refractory multidrug resistant leukemia (67). Inhibition of mTOR is an effective strategy for overcoming chemotherapy resistance. Wu et al. indicated that the mTOR inhibitor CCI-779 can make prostate cancer cells sensitive to docetaxel (68). Although the reversal of drug resistance can be achieved at the cellular level, recent studies have reported the results of phase two clinical trials of dual inhibitors of mTOR in the treatment of metastatic prostate cancer (69). In the end, the patients terminated the experiment because of their inability to tolerate the side effects of the drug. The proposed inhibitors are also not able to completely suppress signal transduction downstream of the pathway, so research in this field is counting on clinical trials of third-generation mTOR inhibitors. Alshaker et al. who used *in vivo* and *in vitro* experiments, demonstrated that the mTOR inhibitor, everolimus, is sensitive to docetaxel. This was done by downregulating HIF-1 and sphingosine kinase 1 (70).

## Discussion

Metabolic reprogramming determines the biological behavior of tumor cells, so that it remains a therapeutic target of choice. As mentioned above, Drug-resistant tumor cells rely on different metabolic pathways, but if the metabolic profile is well established, targeting main bioenergetic pathway altered could improve significantly the outcome of patients. For more effectively eliminate drug-resistant cells, combined strategies involving modulation of both glycolytic and mitochondrial pathways might be a good choice.

In the future, we foresee an era in which malignancies, will be classified on the basis of enhancement or alteration of metabolic pathways, Metabolic symbiosis is rapidly being translated into clinical advances and we anticipate the development of additional novel therapies for drug resistance tumor in the near future.

## Author Contributions

LM: study conception, design, drafting of manuscript, and critical revision. XZ: performed manuscript review. All authors have read and approved the content of the manuscript.

### Conflict of Interest

The authors declare that the research was conducted in the absence of any commercial or financial relationships that could be construed as a potential conflict of interest.

## Abbreviations

ATP: Adenosine Triphosphate
HK: Hexokinase
PK: Pyruvate kinase
PGM: Phosphoglycerate mutase
ENO: Enolase
FBP: Fructose-1,6-Bisphosphatase
LDH: Lactate dehydrogenase
HIF1: Hypoxia-inducible factor 1
TCA: The tricarboxylic acid
OXPHOS: Oxidative phosphorylation
GLUTs: Glucose transporters
PFK: Phosphofructokinase
TIGAR: Tp53-induced glycolysis and apoptosis regulator
SCO2: Synthesis of cytochrome *c* oxidase
PI3K: Phosphatidylinositol-3-kinase
mTOR: Mammalian target of rapamycin
EMT: Epithelial-mesenchymal transition
TsC2: Tuberous sclerosis 2
AMPK: AMP-activated protein kinase
CSC: Cancer stem cell.
